# Detectable Viral Load in Late Pregnancy among Women in the Rwanda Option B+ PMTCT Program: Enrollment Results from the Kabeho Study

**DOI:** 10.1371/journal.pone.0168671

**Published:** 2016-12-22

**Authors:** Michelle M. Gill, Heather J. Hoffman, Emily A. Bobrow, Placidie Mugwaneza, Dieudonne Ndatimana, Gilles F. Ndayisaba, Cyprien Baribwira, Laura Guay, Anita Asiimwe

**Affiliations:** 1 Elizabeth Glaser Pediatric AIDS Foundation, Washington, District of Columbia, United States of America; 2 Department of Epidemiology and Biostatistics, Milken Institute School of Public Health, The George Washington University, Washington, District of Columbia, United States of America; 3 Rwanda Ministry of Health, Kigali, Rwanda; 4 Elizabeth Glaser Pediatric AIDS Foundation, Kigali, Rwanda; 5 University of Maryland, Baltimore, Maryland, United States of America; 6 Rwanda University Teaching Hospitals, Kigali, Rwanda; University of Cincinnati College of Medicine, UNITED STATES

## Abstract

There are limited viral load (VL) data available from programs implementing “Option B+,” lifelong antiretroviral treatment (ART) to all HIV-positive pregnant and postpartum women, in resource-limited settings. Extent of viral suppression from a prevention of mother-to-child transmission of HIV program in Rwanda was assessed among women enrolled in the Kigali Antiretroviral and Breastfeeding Assessment for the Elimination of HIV (Kabeho) Study. ARV drug resistance testing was conducted on women with VL>2000 copies/ml. In April 2013-January 2014, 608 pregnant or early postpartum HIV-positive women were enrolled in 14 facilities. Factors associated with detectable enrollment VL (>20 copies/ml) were examined using generalized estimating equations. The most common antiretroviral regimen (56.7%, 344/607) was tenofovir/lamivudine/efavirenz. Median ART duration was 13.5 months (IQR 3.0–48.8); 76.1% of women were on ART at first antenatal visit. Half of women (315/603) had undetectable RNA-PCR VL and 84.6% (510) had <1,000 copies/ml. Detectable VL increased among those on ART > 36 months compared to those on ART 4–36 months (72/191, 37.7% versus 56/187, 29.9%), though the difference was not significant. The odds of having detectable enrollment VL decreased significantly as duration on ART at enrollment increased (AOR = 0.99, 95% CI: 0.9857, 0.9998, p = 0.043). There was a higher likelihood of detectable VL for women with lower gravidity (AOR = 0.90, 95% CI: 0.84, 0.97, p = 0.0039), no education (AOR = 2.25, (95% CI: 1.37, 3.70, p = 0.0004), nondisclosure to partner (AOR = 1.97, 95% CI: 1.21, 3.21, p = 0.0063) and side effects (AOR = 2.63, 95% CI: 1.72, 4.03, p<0.0001). ARV drug resistance mutations were detected in all of the eleven women on ART > 36 months with genotyping available. Most women were receiving ART at first antenatal visit, with relatively high viral suppression rates. Shorter ART duration was associated with higher VL, with a concerning increasing trend for higher viremia and drug resistance among women on ART for >3 years.

## Introduction

In April 2012, the Rwandan government initiated a policy of lifelong antiretroviral therapy (ART) for all HIV-infected pregnant women for prevention of mother-to-child transmission (PMTCT) regardless of CD4 count or clinical status (also known as Option B+). In 2013, the World Health Organization consolidated antiretroviral (ARV) guidelines endorsed this approach, particularly in HIV-endemic resource-limited settings [1]. These guidelines also promoted the use of viral load (VL) testing as the preferred approach to monitoring ART and identifying treatment failure, recommending testing six months after starting ART and every 12 months thereafter. Per Rwandan national guidelines, pregnant women entering the PMTCT program have a VL test performed every 12 months [2].

Studies in both resource-rich and resource-limited countries have demonstrated a link between duration of ART and risk of infant transmission in HIV-infected pregnant women [3–5], with the lowest rates of transmission observed among women starting ART preconception or early in the first trimester and having undetectable VL at delivery [5–7]. The time to achieve viral suppression varies by VL at treatment initiation. In a study from South Africa, in pregnant women with median pre-ART VL of 10,000 copies/ml, the median time from ART initiation to VL <1000 copies/ml was 24 days and to <50 copies/ml was 94 days [8]. Factors associated with detectable VL at delivery included higher VL levels at ART initiation, <50% adherence to treatment, shorter duration of ART, and receipt of protease inhibitor (PI)-based regimens [8–12].

However, few studies have evaluated the extent of viral suppression since the scale-up of Option B+, particularly in women already on ART at entry into antenatal care. Existing literature addresses pregnant women’s VL outcomes among those initiating ART for their own health, receiving services outside of an Option B+ context, or is limited to ART-naïve women [13–16].

The population of women receiving ART from pre-conception onward will be rapidly increasing with the 2015 WHO recommendations for ART initiation in all HIV-positive individuals regardless of CD4 cell count [17]. Because VL during pregnancy is associated with risk of MTCT, it is important to understand the extent of viral suppression among women entering pregnancy on ART, and factors associated with lack of suppression.

The Elizabeth Glaser Pediatric AIDS Foundation (EGPAF) and the Rwandan Ministry of Health collaborated on the Kigali Antiretroviral and Breastfeeding Assessment for the Elimination of HIV (Kabeho) Study, with a primary aim to evaluate 24-month HIV-free survival in a cohort of infants born to HIV-positive women enrolled in the PMTCT program. Women enrolled in this study underwent viral monitoring at enrollment, delivery, and 18 months and 24 months postpartum. This study allows for an assessment of the extent of viral suppression during pregnancy in women who received ART prior to or during pregnancy, with many women already receiving ART at their first antenatal care (ANC) visit. We present these results from women at the time of study enrollment, with an examination of factors associated with detectable maternal VL in late pregnancy or early postpartum.

## Materials and Methods

### Study Design

The Kabeho Study was an observational prospective cohort of 608 HIV-positive eligible women enrolled during the third trimester of pregnancy or within two weeks postpartum from PMTCT programs, and their infants, who were followed until the children reached approximately 24 months of age. The sample size calculation was based on the overall study’s primary endpoint of HIV-free survival at the end of the follow-up period.

### Study Population

Between April 2013 and January 2014, eligible pregnant and postpartum women in 14 Kigali antenatal clinics with at least 50 HIV-positive women per year, were referred consecutively by clinic staff until the site-specific target was reached. Eligibility criteria included documented HIV infection, being in the third trimester of pregnancy or within two weeks post-delivery, participation in the PMTCT program during ANC at one of the study sites, planning to remain in the Kigali area after delivery, and able and willing to provide informed consent for herself and her infant. Per ethical guidelines in Rwanda, informed consent was also required from parents of women less than 18 years of age unless the women were legally married. Women presenting early in pregnancy could be re-screened at a subsequent visit once they reached the third trimester or were within two weeks of delivery.

### Data Collection

Women underwent an enrollment interview, which included demographic information, HIV and ART-related history, ARV adherence, ARV side effects, nutritional information, and infant feeding plans. Adherence was assessed through self-reported recall of doses missed in the past three days. Medical and laboratory information were also collected from the relevant facility records. Responses were documented on paper forms. Data were reviewed for completeness and quality, and entered into the study database using a data capture system called SurveyCTO (V2.10; Dobility, 2016). RNA-PCR testing for VL in women was done at the time of enrollment by the National Reference Laboratory (NRL) using the Roche COBAS Ampliprep/TaqMan (V2.0). ARV drug resistance testing was conducted on women with VL>2000 copies/ml at enrollment. Testing was done at the NRL using the FDA-licensed ViroSeq genotyping kits and a 3500 XL ABI genetic analyzer according to manufacturer instructions. As standard of care, CD4 testing was conducted for all women testing HIV-positive in ANC and then every six months thereafter. The most recent CD4 test result was abstracted from women’s medical records at the time of enrollment.

All women provided written informed consent to participate in this minimal risk study. All study staff received training on data collection and on research with human subjects. Ethical approvals were obtained from the Rwandan National Ethics Committee, the Rwanda National Health Research Committee, and the George Washington University Institutional Review Board. The protocol was registered with the U.S. National Institutes of Health at clinicaltrials.gov (No: NCT02295800).

### Data Analysis

Descriptive summary statistics were calculated for predictor and outcome variables of interest using frequencies and percentages for categorical variables, and medians and interquartile ranges for continuous variables. The laboratory determination of “undetectable” for HIV VL was used, defined as <20 copies/ml, the lower limit of detection with the laboratory assay. To examine the likelihood of detectable VL in pregnant and postpartum Kabeho Study participants, unadjusted and adjusted odds ratios (OR) for detectable VL were estimated with corresponding 95% confidence intervals using generalized estimating equations (GEE) having a binomial distribution with a logit link function and a compound symmetry working correlation structure. A sensitivity analysis was conducted to compare women with and without VL detection by dividing the sample into the two groups, women who were already on ART at first ANC visit and women who were initiated on ART in ANC. Additional sensitivity analyses were conducted to determine if the odds of detectable VL changed significantly in the adjusted model when women enrolled postpartum women were excluded or those with missing VL results were considered virally suppressed. Potential factors associated with detectable VL were selected based on the existing literature; any women without ART duration information were excluded. Among women with genotyping data, the likelihood of ARV drug resistance was assessed using GEE. ART duration, viral load, age at enrollment, and all two-way interactions were examined in these models and unadjusted and adjusted OR for resistance were estimated with corresponding 95% confidence intervals.

All p-values were adjusted for multiple comparisons using the Tukey-Kramer method. All statistical tests were two-sided and the level of statistical significance was set at 0.05. Statistical analyses were conducted using SAS^®^ 9.3 (Cary, North Carolina).

## Results

### Study Population

**Fig 1:**
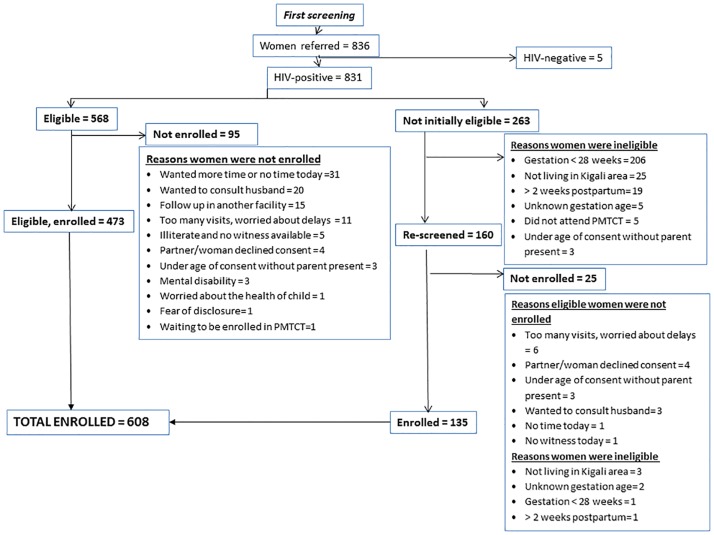
Kabeho Study Cohort Screening and Enrollment Diagram.

A total of 831 HIV-positive women were referred to study staff for screening (Fig 1). Of these, 263 women were ineligible and 95 were eligible, but did not enroll. The majority of women who declined participation cited needing more time to consider study participation (n = 31) or wanting to consult their husbands (n = 20). Of the 160 HIV-positive women re-screened at a subsequent clinic visit, 135 were eligible and enrolled. The final number enrolled was the target of 608, though one woman did not complete the enrollment interview; 15.1% (n = 92) joined within two weeks postpartum.

Table 1 presents characteristics for HIV-positive pregnant and postpartum women at enrollment overall and by baseline detectable VL (47.8%, n = 288/603) and undetectable VL (52.2%, n = 315/603). Five women were missing VL at enrollment. Women had a median age of 29 years (IQR 25–34) and the majority (79.1%, n = 481) were married or co-habiting. Most women (60.2%, n = 366) had attended only primary school.

**Table 1:** Characteristics of HIV-Infected Pregnant and Postpartum Women at Study Enrollment (N = 608)*.

		Women by detectable and undetectable VL^+^		
Characteristic	Total^#^	Detectable viral load (≥20 copies/ml)	Undetectable viral load (<20 copies/ml)	Total
**Age, n, median years (IQR)**	**608, 29 (25–34)**	**28 (24–33)**	**30 (26–34)**	
≤19, n (%)	19 (3.1)	11 (57.9)	8 (42.1)	19
20–<24	120 (19.7)	72 (60.0)	48 (40.0)	120
25–<30	184 (30.3)	90 (48.9)	94 (51.1)	184
30–<35	155 (25.5)	57 (37.0)	97 (63.0)	154
>35	130 (21.4)	58 (46.0)	68 (54.0)	126
**Gravidity, n, median (IQR)**	**607, 3 (2–4)**	**3 (2–4)**	**3 (2–4)**	
1, n (%)	104 (17.1)	61 (58.7)	43 (41.3)	104
2–4	392(64.6)	178 (45.6)	212 (54.4)	390
≥ 5	111 (18.3)	49 (45.0)	60 (55.0)	109
**Current marital status, n (%)**	**608**	**288**	**315**	**603**
Married or co-habitating	481 (79.1)	216 (45.3)	261 (54.7)	477
Never married	62 (10.2)	37 (59.7)	25 (40.3)	62
Divorced/Separated/Widowed	65 (10.7)	35 (54.7)	29 (45.3)	64
**Educational attainment, n (%)**	**608**	**288**	**315**	**603**
No education	100 (16.4)	53 (53.5)	46 (46.5)	99
Attended primary school	366 (60.2)	177 (48.8)	186 (51.2)	363
Attended secondary school and above	142 (23.4)	58 (41.1)	83 (58.9)	141
**Timing of enrollment visit, n (%)**	**607**	**288**	**315**	**603**
3^rd^ trimester	515 (84.8)	237 (46.3)	275 (53.7)	512
Within 2 weeks postpartum	92 (15.2)	51 (56.0)	40 (44.0)	91
**Reported HIV status of father of the child, n (%)**	**608**	**288**	**315**	**603**
HIV-positive	306 (50.3)	141 (46.5)	162 (53.5)	303
HIV-negative	192 (31.6)	83 (43.5)	108 (56.5)	191
Unknown	110 (18.1)	64 (58.7)	45 (41.3)	109
**Disclosed HIV status to, n (%)**	**607**	**288**	**315**	**603**
Partner	497 (81.9)	219 (44.4)	274 (55.6)	493
Someone other than partner	83 (13.7)	53 (63.9)	30 (36.1)	83
No one	27 (4.4)	16 (59.3)	11 (40.7)	27
**Time since HIV diagnosis, n, median months (IQR)**	**566, 38.0 (4.7–83.5)**	**274, 23.5 (2.6–76.4)**	**288, 48.6 (16.8–89.9)**	
<4 months, n (%)	122 (20.1)	95 (77.9)	27 (22.1)	122
>4 months– 1 year	69 (11.3)	31 (45.6)	37 (54.4)	68
>1–3 years	87 (14.3)	32 (37.2)	54 (62.8)	86
>3–5 years	71 911.7)	26 (37.1)	44 (62.9)	70
> 5 years	217 (35.7)	90 (41.7)	126 (58.3)	216
Unknown time	42 (6.9)	14 (34.1)	27 (65.9)	41
**Time on ART, n, median months (IQR)**	**594, 13.5 (3.0–48.8)**	**277, 3.6 (1.6–38.4)**	**313, 23.9 (4.8–57.2)**	
Not on ART	13 (2.1)	11 (84.6)	2 (15.4)	13
<4, n (%)	214 (35.3)	149 (70.3)	63 (29.7)	212
>4–12	73 (12.0)	24 (32.9)	49 (67.1)	73
>12–24	62 (10.2)	17 (27.4)	45 (72.6)	62
>24–36	53 (8.7)	15 (28.8)	37 (71.2)	52
>36	192 (31.6)	72 (37.7)	119 (62.3)	191
**Time on current ART regimen, n, median months (IQR)**	**594, 8.8 (2.3–34.9)**	**277, 2.8 (1.0–22.8)**	**313, 20.2 (4.3–39.2)**	
Not on ART	13 (2.1)	11 (84.6)	2 (15.4)	13
<4, n (%)	240 (39.5)	165 (69.3)	73 (30.7)	238
>4–12	77 (12.7)	24 (31.2)	53 (68.8)	77
>12–24	69 (11.4)	19 (27.5)	50 (72.5)	69
>24–36	64 (10.5)	18 (28.6)	45 (71.4)	63
>36	144 (23.7)	51 (35.7)	92 (64.3)	143
**On ART at first visit, n (%)**	**607**	**288**	**315**	**603**
Yes	462 (76.1)	193 (42.0)	267 (58.0)	460
No	145 (23.9)	95 (66.4)	48 (33.6)	143
**Current ART regimen, n (%)**	**607**	**288**	**315**	**603**
ART with TDF + 3TC + EFV	344 (56.7)	186 (54.4)	156 (45.6)	342
ART with AZT + 3TC + NVP	62 (10.2)	26 (41.9)	36 (58.1)	62
ART with TDF + 3TC + NVP	139 (22.9)	41 (29.5)	98 (70.5)	139
ART with a different regimen	48 (7.9)	24 (51.1)	23 (48.9)	47
None	14 (2.3)	11 (84.6)	2 (15.4)	13
**Previous exposure to another regimen before current one, n (%)**	**214**	**87**	**125**	**212**
PMTCT for a previous pregnancy only	113 (52.8)	49 (43.8)	63 (56.2)	112
PMTCT + HIV treatment with a different drug regimen	17 (7.9)	7 (43.8)	9 (56.2)	16
HIV treatment with a different drug regimen only	72 (33.6)	26 (36.1)	46 (63.9)	72
Other reason	12 (5.6)	5 (41.7)	7 (58.3)	12
**Adherence in previous 3 days, n (%)**	**592**	**275**	**313**	**588**
Took all doses in last 3 days	538 (90.9)	246 (46.1)	288 (53.9)	534
Took some doses in last 3 days	46 (7.8)	24 (52.2)	22 (47.8)	46
Took no doses in last 3 days	8 (1.4)	5 (62.5)	3 (37.5)	8
N/A	15	13	2	15
**Current side effects reported in the last month, n (%)**^	**105**	**71**	**34**	**105**
Dizziness	56 (53.3)	38 (67.9)	18 (32.1)	56
Gastrointestinal problems	40 (38.1)	27 (67.5)	13 (32.5)	40
Trouble sleeping/nightmares	27 (25.7)	19 (70.4)	8 (29.6)	27
Skin problems	15 (14.3)	10 (66.7)	5 (33.3)	15
Other	24 (22.9)	15 (62.5)	9 (37.5)	24
**CD4 test results (cells/mm3), n (%)**	**538**	**240**	**296**	
<200	32 (5.9)	21 (65.6)	11 (34.4)	32
200 to <350	106 (19.7)	58 (54.7)	48 (45.3)	106
350 to <500	161 (29.9)	67 (41.6)	94 (58.4)	161
>500	239 (44.4)	94 (39.7)	143 (60.3)	237
NA	67	46	19	65

3TC: lamivudine; ART: antiretroviral therapy; AZT: zidovudine; EFV: efavirenz; NVP: nevirapine; PMTCT: prevention of mother-to-child HIV transmission prophylaxis; TDF: tenofovir disoproxil fumarate.

*There is one woman with unknown viral load for whom many variables are missing as she did not complete the enrollment interview.

^#^ Total is inclusive of those with unknown VL (607 or 608) and percentages are calculated vertically in this column.

^+^Percentages are calculated for detectable versus undetectable VL and the total excludes the five women with unknown VL.

^Women could report more than one side effect, so the numbers of women responding with any side effect are also included by detectable and undetectable VL.

### HIV and Antiretroviral Treatment History

Table 1 also includes information on HIV diagnosis and disclosure, previous ART exposure and adherence to ARVs. Half of the women reported that the father of the baby was also HIV-positive (50.3%, n = 306), though a partner discordancy rate of 31.6% (n = 192) was also found. Many women (81.9%, n = 497) had disclosed to their partners, often in addition to someone else. Women had known their HIV infection status for a median of 38.0 months (IQR 4.7–83.5).

Although all study participants were enrolled in the PMTCT program per the eligibility criteria, 13 women were not on ART at study entry, three of whom were enrolled postpartum. Reasons included provider postponing treatment initiation until serum creatinine test results were returned (n = 4), women who had previously been lost to follow-up (n = 4), drug stock-out (n = 1), refusal to take ARVs (n = 1), and not yet initiated without specific reason cited (n = 3).

Women were on ART for a median of 13.5 months (IQR 3.0–48.8), with the median time on their current regimen being 8.8 months (IQR 2.3–34.9); 76.1% (n = 462) of women were already on ART at their first ANC visit. Of those not on ART at enrollment, 58.6% (n = 85/145) were first diagnosed as HIV-positive at their first ANC visit. The most common ARV regimen (56.7%, n = 344) was tenofovir (TDF), lamivudine (3TC), and efavirenz (EFV). Overall, 35.3% (n = 214) of women reported taking another regimen previously, with 21.4% (n = 130) reporting having received a PMTCT regimen (mono, combination or triple therapy) during a prior pregnancy. Of those currently on ART, 90.9% (n = 538/592) reported taking all of their doses in the past three days. Side effects were reported in the past month by 17.3% (n = 105/607) of women, with dizziness as most common (n = 56), followed by gastrointestinal problems (n = 40), trouble sleeping/nightmares (n = 27), and skin problems (n = 15).

### Viral Load

**Fig 2:**
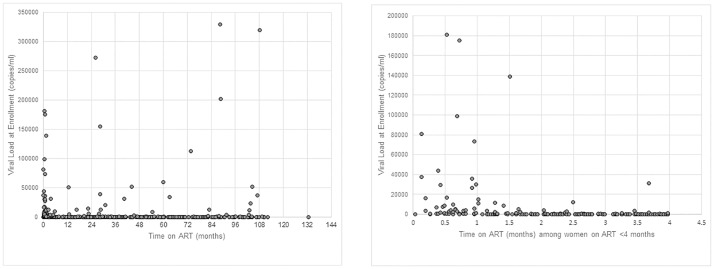
Distribution of viral load results at enrollment by time on ART. (A) Time on ART for all study women with duration and VL information available. (B) Time on ART only for study women on ART < 4 months with VL results available. Both figure parts exclude one woman with an outlying VL result of 900,000 copies/ml and <1 month on ART for ease of viewing.

Half of women (52.2%, n = 315/603) had undetectable VL at study enrollment. Overall, 84.6% (n = 510) had a VL of less than 1,000 copies/ml. Fig 2A shows the distribution of enrollment VL results by ART duration as a continuous variable (in months). VL was undetectable in 15.4% (n = 2/13) women not on ART at enrollment, 29.7% (n = 63/212) of those on ART for ≤4 months, and 66.1% (n = 250/378) of those on ART for >4 months. Detectable VL at enrollment was observed in 37.7% (n = 72/191) of women on ART for >36 months compared to 29.9% (n = 56/187) of women on ART for 4–36 months (AOR = 0.70, 95% CI: 0.45–1.10, p = 0.12). Overall, 65.9% (n = 201/305) of women on ART for more than 12 months achieved undetectable VL at enrollment with 89.8% (n = 274/305) of women reaching a VL of less than 1,000 copies/ml.

Fig 2B illustrates in more detail the distribution of enrollment VL by time on ART from 0–4 months, as some women had initiated ART only a short time before the enrollment blood draw. The percentage of women with undetectable VL increased from 4.1% (n = 2/49) in women on ART <1 month to 55.9% (n = 33/59) in women on ART for 4 months, with a concomitant gradually sloped downward trend in those with detectable VL.

There were 288 women with detectable enrollment VL in this sample. After adjusting for the other explanatory variables, the odds of having detectable VL were significantly lower for women with more pregnancies (AOR = 0.90, 95% CI: 0.84, 0.97, p = 0.0039). The odds of having detectable VL for those with no education was 2.25 times the odds for those who attended secondary school or above (95% CI: 1.37, 3.70, p = 0.0004) (Table 2). Women who did not disclose their HIV status or disclosed to someone other than their partner were more likely to have detectable VL compared to those who disclosed to their partner (AOR = 1.97, 95% CI: 1.21, 3.21, p = 0.0063). The adjusted odds of having detectable VL for those reporting ART side effects was 2.63 times the odds for those not reporting side effects (95% CI: 1.72, 4.03, p<0.0001). The odds of having detectable enrollment VL decreased significantly as duration on ART at enrollment increased (AOR = 0.99, 95% CI: 0.9857, 0.9998, p = 0.043).

**Table 2:** Unadjusted and adjusted odds ratios from GEE models—examining the likelihood of detectable viral load at enrollment in pregnant and postpartum Kabeho Study participants.

		Unadjusted		Adjusted*	
Explanatory variables	N	Odds ratios (95% CI)^#^	P-value	Odds ratios (95% CI)	P-value
**Maternal age**	603	0.97 (0.94, 0.99)	0.015	1.01 (0.98, 1.04)	0.50
**Gravidity**	603	0.88 (0.79, 0.97)	0.012	0.90 (0.84, 0.97)	0.0039
**Educational attainment**	603		0.012		0.0005
No education		1.67 (1.08, 2.60)	0.018	2.25 (1.37, 3.70)	0.0004
Attended primary school		1.37 (0.87, 2.16)	0.25	1.57 (0.96, 2.56)	0.080
Attended secondary school and above		REF	REF	REF	REF
**HIV status of father of the child**	603		0.0006		0.45
HIV-positive		0.61 (0.42, 0.89)	0.0065	1.11 (0.65, 1.90)	0.90
HIV-negative		0.54 (0.35, 0.83)	0.0020	0.86 (0.42, 1.76)	0.87
Unknown		REF	REF	REF	REF
**Disclosed HIV status to partner**	603		<0.0001		0.0063
Yes		REF	REF	REF	REF
No		2.11 (1.51, 2.95)	<0.0001	1.97 (1.21, 3.21)	0.0063
**Time on antiretroviral therapy (ART)**	590	0.9892 (0.9841, 0.9942)	<0.0001	0.9927 (0.9857, 0.9998)	0.043
**Adherence**	588		0.28		0.37
≥95% adherence		REF	REF	REF	REF
<95% adherence		1.36 (0.78, 2.38)	0.28	1.32 (0.72, 2.43)	0.37
**Side effects**	573		<0.0001		<0.0001
Present		3.00 (2.07, 4.36)	<0.0001	2.63 (1.72, 4.03)	<0.0001
Absent		REF	REF	REF	REF

*The adjusted model included all variables listed in this table.

^#^All 95% CIs have been adjusted for multiple comparisons using the Tukey-Kramer method.

In the sensitivity analysis stratified by ART initiation prior to pregnancy versus during pregnancy, there was a non-significant trend for duration of ART to be associated with detectable VL at enrollment in women on ART prior to pregnancy (AOR = 0.9943, 95% CI: 0.9864, 1.0022, p = 0.16). For women first starting ART during pregnancy, ART duration was significantly associated with having detectable enrollment VL (AOR = 0.43, 95% CI: 0.29, 0.63, p<0.0001).

For the other sensitivity analyses, the significance of all variables remained the same in the adjusted model, regardless of whether we assumed the five women (0.8%) with missing VL were detectable or excluded the 91 women (15.0% of the sample) enrolled within two weeks post-delivery.

### Antiretroviral Drug Resistance

**Fig 3:**
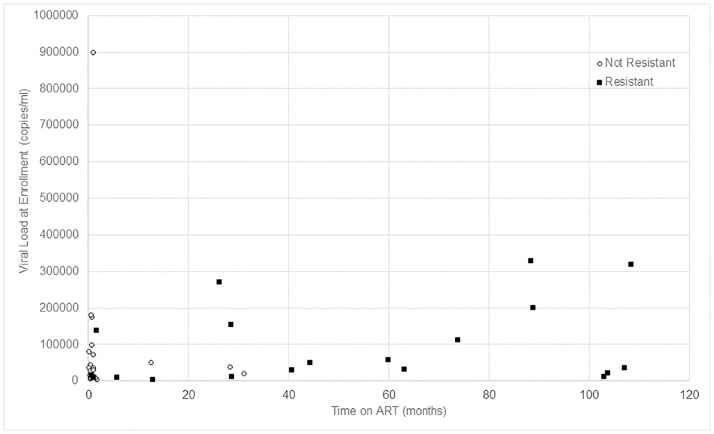
Distribution of viral load results at enrollment by time on ART among women with and without HIV drug resistance (major NRTI and NNRTI mutations only).

There were 80 women (13.3%) with VL >2,000 copies/ml for genotyping for ARV drug resistance mutations. Genotyping results were available for 57.5% (n = 46/80) of these women, with mutations associated with ARV resistance detected in 63.0% (n = 29/46). Major nucleoside reverse transcriptase inhibitors (NRTI) and/or non-nucleoside reverse transcriptase inhibitors (NNRTI)-associated mutations were detected in 19 women (41.3%). Nine women had minor PI mutations that may be normal variants in non-clade B HIV subtypes and one women had a low level NNRTI mutation [18]. Information on duration of ART was available for 89.1% (n = 41/46) of women, including all the women with resistance mutations detected. Fig 3 presents the data on the detection of major NRTI and NNRTI mutations in women by their VL and time on ART. Major resistance mutations were detected in 13.6% (n = 3/22) of women on ART for ≤4 months, 62.5% (n = 5/8) of women with ART duration of 4–36 months, and in all 11 women on ART for >36 months. All 11 women had NNRTI mutations; six had NNRTI mutations alone, three had NNRTI plus thymidine analogue mutations (TAMs), and two had NNRTI plus non-TAM NRTI mutations.

There were significant interactions between VL and both duration of ART (p = 0.026) and maternal age (p = 0.023) when evaluating the likelihood of drug resistance at enrollment into the study. For example, in women with an enrollment VL of 100,000 copies/ml, the odds of resistance was significantly higher for those with a longer ART duration (AOR = 1.11, 95% CI: 1.04, 1.18, p = 0.0012), and the odds of resistance was significantly lower for those who were older (AOR = 0.33, 95% CI: 0.14, 0.75, p = 0.0086).

## Discussion

The Kabeho Study demonstrated the success of a national PMTCT program implementing Option B+ in an African urban setting. Relatively high rates of VL suppression, partner disclosure of HIV status and ART adherence were found one year after implementation of Option B+ in Rwanda. In Kigali, 76% of women entering ANC were already receiving ART. Overall, 52% of women had undetectable VL, with 85% having VL <1000 copies/ml. As expected, detectable VL was associated with a shorter duration of ART. However, there was a trend towards increasing VL after 36 months on treatment with the concomitant detection of major NRTI and NNRTI mutations.

Shorter duration of ART has been associated with higher risk of detectable VL in a number of studies [8, 11–12, 19]. The median time to reach viral suppression, defined as <50 copies/ml, in a study of South African women initiating EFV-based ART was 13.7 weeks, although this varied by pre-ART VL [8]. In a study from the US, using a VL of <400 copies/ml as the threshold for detectability, the median number of days to achieve viral suppression were similar between ARV-naïve and ARV-experienced cohorts of pregnant women, at 25 and 27 days, respectively [12]. PI-based ART has been associated with slower viral suppression in some studies from developed countries [11–12]; however, we could not assess this as EFV-based ART was used in the Rwanda, as recommended by the WHO guidelines [1].

The increasing proportion of women on ART for greater than 36 months with detectable VL and ART resistance late in pregnancy or around the time of delivery is concerning. This is consistent with data from Swaziland where increasing time on ART was positively associated with detectable VL in adult patients on ART for more than six months, with a median ART duration of 2.8 years [20]. This highlights the importance of initiation of ongoing VL monitoring within PMTCT and ART programs. Though small numbers, all women on ART three or more years with available genotyping results had NRTI and/or NNRTI resistant mutations detected. Stadeli and Richman found rates of acquired drug resistance in resource-limited settings steadily increased with time on ART ranging from 7.2% for patients on ART for 6–11 months to up to 20% detectable resistance mutations for those on ART for 36 months or longer [21]. Detection of major mutations in 41.3% of women in our study is consistent with other studies of women 1–2 years postpartum with virological failure and levels of resistance ranging from 34% to 46% [15,22–23]. However, we assessed resistance among antenatal women with variable but longer ART durations and a higher RNA threshold for genotyping evaluation (2,000 versus 1,000 copies/ml).

Women reported a high rate of disclosure of their HIV status to partners in our study. These high rates among study women may be related to the emphasis in Rwanda on partner testing [2]. It was a significant protective factor for reaching undetectable VL at enrollment, when compared to women who either disclosed to someone other than their partner or to no one, highlighting the importance of discussing one’s status with the child’s father and suggesting that disclosure alone does not confer this same benefit. Lack of disclosure of HIV status to partners or others was associated with viral failure in adults on ART ≥6 months in rural Lesotho and pregnant women enrolled in the French Perinatal Cohort [24–25].

Adherence was not a significant predictor of detectable VL at enrollment in our logit model. The questionnaire only captured self-reported missed doses within the last three days, which may not have been a robust measurement and highlights the need for better measures of adherence in the clinic setting. However, a three-day measure of adherence was used in the Kabeho Study to be consistent with previous research conducted in Rwanda, which found a similar ART adherence rate among women of 91.0% [26]. There were also low numbers of women who did not adhere, which would make it difficult to detect a significant difference. However, detectable VL was associated with women who reported ARV side effects in the past month, which may reflect poor adherence among this group. Among HIV-positive pregnant women on ART at the time of their first ANC visit in South Africa, having a VL greater than 1000 copies/ml was associated with missing ≥3 doses in the past 90 days [8].

Viral suppression rates using the assay’s lower limit of detection of 20 copies/ml, are presented along with the full range of VL results stratified by time on ART. While the exact VL threshold associated with an elevated risk of mother-to-child transmission is not well defined, very low rates of infant transmission can be achieved when maternal VL is suppressed at delivery [5].

The current WHO and Rwandan virological criterion for treatment failure is 1,000 copies/ml or more on two separate tests [1,2]. Therefore, we were not able to assess treatment failure at enrollment from our single VL measurement. In Rwanda, guidelines for management of individuals with VL >1,000 copies/ml, including pregnant women, includes two repeat measurements at three-month intervals, additional adherence counseling and if results do not improve, referral for clinical consultation regarding need for second-line therapy. However, in pregnant women, such management may result in women delivering before viral suppression has been achieved. In our study, nearly 50% of women had detectable VL at enrollment in the third trimester/two weeks postpartum, putting their infants at higher risk of infection. Women with ≤4 months of ART were at greater risk of having detectable VL and the sensitivity analysis demonstrated the importance of ART duration in terms of VL detection among women first starting ART during pregnancy as time to suppression will be longer for women with higher VL.

Ensuring women are tested and retested for HIV during pregnancy according to national guidelines in order to initiate ART as soon as possible after diagnosis is a key step in the PMTCT cascade. In our study, 13 women were not on ART at the time of enrollment. While we would expect these women to have detectable VL, it was important to include them in this real-world description of a maturing Option B+ program as lack of treatment is an important factor in determining detectable VL. Early attendance at ANC to allow maximum time on ART for those newly initiating is also critical. Additionally, management of HIV-positive pregnant women with more frequent VL testing and ARV resistance testing, as more asymptomatic women are initaited on ART, would be advisable. For women with detectable or very high VL near delivery, administering more intensive infant ARV prophylaxis to further minimize risk of intrapartum/early peripartum transmission could be considered, which has been found to be effective in reducing intrapartum transmission in infants born to women who have not received ARV prophylaxis during pregnancy [27].

This study has some limitations. While the findings highlight several successful aspects of the program, all study facilities are high-volume sites located in Kigali. The study does not include any sites based in rural areas, or urban areas other than Kigali. Given that women enrolled in the national PMTCT program in areas outside Kigali may differ in their levels of awareness, social norms and access to services, this is a limitation of the study in terms of making inferences from the study population to the overall population in Rwanda. In addition, because women were enrolled later in pregnancy or early postpartum (necessitated by the timing of award funding), there is a lack of first ANC visit data for all participants and pre-ART initiation data for those newly initiated. However, the Kabeho Study fulfills a critical need for evaluation of VL suppression data from ART-experienced and ART-naïve women in ANC in an established Option B+ program. The study presents a range of socio-demographic, clinical and ART-related factors that were examined as potential predictors of detectable VL within the context of routine VL monitoring in one of the early Option B+ adopting countries.

This study found high rates of viral suppression in a PMTCT Option B+ program in which the majority of women were already receiving ART at their first antenatal care visit. While VL decreased the longer women were on treatment, the increasing trend in higher VL with longer ART duration demonstrates the importance of continued monitoring, particularly in women who have subsequent pregnancies. The enrollment VL results of the Kabeho Study reflect the reality of a real world setting in one of the earliest national programs using Option B+. Future study results on continued VL monitoring in the breastfeeding period and resistance testing for those with high VL will further our understanding on optimal approaches to elimination of pediatric AIDS.
